# Antibiotics in Food Chain: The Consequences for Antibiotic Resistance

**DOI:** 10.3390/antibiotics9100688

**Published:** 2020-10-13

**Authors:** Shashi B. Kumar, Shanvanth R. Arnipalli, Ouliana Ziouzenkova

**Affiliations:** Department of Human Sciences, The Ohio State University, Columbus, OH 43210, USA; kumar.864@osu.edu (S.B.K.); arnipalli.1@buckeyemail.osu.edu (S.R.A.)

**Keywords:** microbiome, resistome, horizontal evolution, quorum-sensing

## Abstract

Antibiotics have been used as essential therapeutics for nearly 100 years and, increasingly, as a preventive agent in the agricultural and animal industry. Continuous use and misuse of antibiotics have provoked the development of antibiotic resistant bacteria that progressively increased mortality from multidrug-resistant bacterial infections, thereby posing a tremendous threat to public health. The goal of our review is to advance the understanding of mechanisms of dissemination and the development of antibiotic resistance genes in the context of nutrition and related clinical, agricultural, veterinary, and environmental settings. We conclude with an overview of alternative strategies, including probiotics, essential oils, vaccines, and antibodies, as primary or adjunct preventive antimicrobial measures or therapies against multidrug-resistant bacterial infections. The solution for antibiotic resistance will require comprehensive and incessant efforts of policymakers in agriculture along with the development of alternative therapeutics by experts in diverse fields of microbiology, biochemistry, clinical research, genetic, and computational engineering.

## 1. Introduction and Background

**Figure 1:**
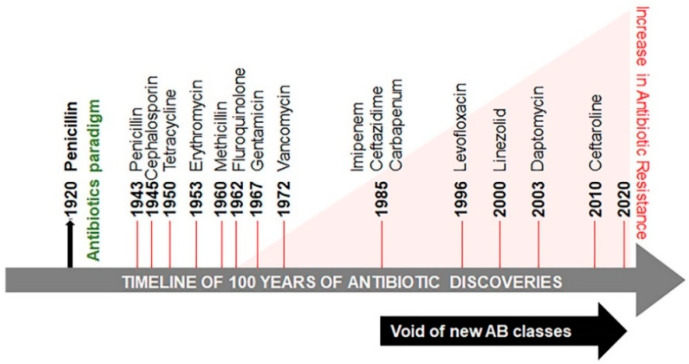
Timeline of antibiotic discovery and its onset of resistance. The antibiotic paradigm emerges out of the followed discovery of penicillin. Between the 1960s and the 1980s there was a surge in the discovery of antibiotics, but this development declined between the 1980s and the 1990s. The identification of new antibiotic classes by pharmaceutical companies has stagnated since 1987 and coincided with progressively increased antibiotic resistance and mortality related to antibiotic-resistant infections.

In 1928 Alexander Fleming serendipitously discovered penicillin [1] (Figure 1). Its utility as medicine became clear following the extraction of a small amount of penicillin from a fungus *Penicillium chrysogenum*, by Howard Florey and Ernst Chain in 1941, at the Radcliffe Infirmary. This extract was initially used for treating a policeman in Oxford, England who contracted a likely infection of *Staphylococcus aureus* with an admixture of various *Streptococci*. The condition of the policeman was initially improved; however, the amount and quality of penicillin synthesis were inadequate at the time. Eventually, sepsis relapsed and the policeman died. Presently, penicillin and other antibiotics are produced in copious amounts. The term antibiotic is defined as a natural or synthetic chemical inhibiting both the growth and survival of microorganisms. Among these antibiotics, methicillin is considered to be one of the most effective. However, studies revealed that sepsis cases increased from 621,000 to 1,141,000 between the years of 2000 and 2008 [2]. The death toll from sepsis rose from 154,000 to 207,000 cases. The extent of this rise is attributed to the emergence of methicillin resistant *S. aureus* (MRSA). MRSA marks the beginning of the development of antibiotic–resistant microbes (also called ESKAPE pathogens, standing for *Enterococcus faecium, S. aureus, Klebsiella pneumoniae, Acinetobacter baumannii, Pseudomonas aeruginosa,* and *Enterobacteriaceae*) [3]. It is reported that in the United States, India, Thailand, and European Union, antibiotic resistance causes more than 23,000, 58,000, 38,000 and 25,000 deaths per year, respectively [4,5,6,7]. The predicted deaths from drug-resistant microbial pathogens could rise from approximately 700,000 per year to 10 million deaths per year by 2050 and threaten global health [8].

Microorganisms are able to develop antibiotic-resistant genes to enhance their survival, thus minimizing the treatment options for microbial infections and increasing mortality in human populations. Antibiotic resistance is classified into three categories based on the threat: urgent, serious, and concerning (Table 1). The global threat of resistance to imipenem antibiotics in *A. baumannii* infections has been reported in both Organization for Economic Co-operation and Development (OECD) and non-OECD countries across the globe [9]. Several reasons are responsible for the development of antibiotic resistance globally and in developing countries, such as India [10,11]. Poor public health conditions and health care systems, availability of antibiotics over the counter, lack of public knowledge of appropriate dosage of antibiotics and their haphazard use, as well as a high incidence of infectious diseases have been proposed as the major factors augmenting the problem. This continuum of antibiotic resistance concept was proposed to describe the progressive interconnecting influence of human, industrial, agricultural, and wild environments [12]. The crude mortality due to infectious diseases in India is 416.75 per 100,000 persons, which is twice the rate in the United States (roughly 200 per 100,000 persons) [13]. The problem is aggravated further by the void in the development of new classes of antibiotics since 1990 (Figure 1) [14,15,16,17].

The advances in social and medical fields, including cancer therapy and organ transplantation, would not have been possible without effective antibiotic treatment to control bacterial infections. However, global antibiotic resistance is on the rise. In this review, we thoroughly apprise comprehensive evidence of various factors leading to the development of antibiotic resistance, followed by route of entry of drug-resistant pathogens into the food chain, and a plethora of alternative strategies to mitigate the menace of antibiotic resistance for a healthier future.

## 2. Drug Resistance Continuum

**Figure 2:**
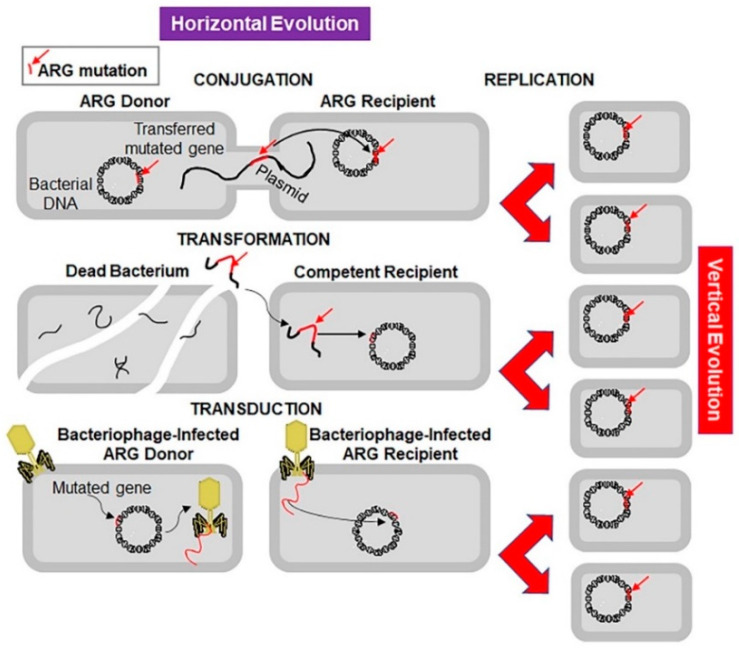
Mechanisms of horizontal and vertical transmission in bacteria for the development of antibiotic resistance. The left panel shows the horizontal transmission of an antibiotic resistant gene (ARG, red line indicated by a red arrow) by the three main mechanisms: conjugation, transformation, and transduction. Conjugation involves transfer of the ARG from a donor bacterium to a recipient by direct contact and plays a crucial role in dissemination of antibiotic resistance. Transformation involves uptake of the free DNA with the ARG from the environment. Transduction is a virus-mediated gene transfer by bacteriophages. The right panel shows the vertical evolution carried out by replication of bacteria containing the ARG.

Microorganisms are evolving rapidly to endure and proliferate in unfavourable environments. Although antibiotic resistance appeared soon after clinical use of antibiotics, initially the problem was of low concern and was condoned (Figure 1) [18]. Sulphonamide-resistant *Streptococcus pyogenes* appeared in the human clinical settings in early 1930s, while penicillin-resistant *S. pyogenes* was noted in the 1940s. The problem of multidrug-resistant enteric bacteria became noticeable in the 1950s [19]. Antibiotic resistance develops as a result of vertical or horizontal evolution (Figure 2). Advantageous mutations cause antibiotic tolerance, which is transmitted to offspring (vertical evolution) or to another bacteria via conjugation, transduction, or transformation mode (horizontal evolution), that are then passed down to progeny (vertical evolution). The comprehensive genomic insights into human pathogens have shown that horizontal gene transfer is an important mechanism of antibiotic resistance gene (ARG) acquisition among microorganisms along with the vertical transfer [20]. A decade ago, an ARG, the New Delhi metallo-β-lactamase 1 (NDM-1) was identified in single isolates of *K. pneumonia* and *Escherichia coli*. Both were isolated from a patient first admitted to a hospital in New Delhi, India, and then repatriated to Sweden [21]. This was followed by the spread of antibiotic resistance in every geographical region [22,23]. NDM-1 has no detectable sequence homology with other classes of these genes, thus indicating their archaic origin [24,25]. Bacteria carrying extended spectrum β-lactamases (ESBL) impart resistance to penicillin and cephalosporins, extensively drug-resistant (XDR) *Mycobacterium tuberculosis*, and multi-drug resistant *A. baumanni*, *Enterobacteriaceae*, *Neisseria gonorrhoea*, and *P. aeruginosa* [26,27].

## 3. The Detrimental Effects of Antibiotics Misuse

The dogma that antibiotics are safe for humans has been dominant for many decades, and only recently has it started to be challenged. Antibiotics are recommended to humans based on rigorous clinical trials, examining antibiotics use against microorganisms, and their efficacy and safety. However, antibiotics can have serious side effects in human cells. The effects of antibiotics on pathways in humans are listed in Table 2 [28]. Some medical professionals and some regulatory agencies continue to underestimate the debilitating effects of antibiotics in humans. For instance, fluoroquinolones are routinely prescribed by medical doctors worldwide, even though they cause several side effects, encompassing damage to muscles, tendons, neuropsychiatric disorders, and mitochondrial toxicity. Given the repeated incidences of fluoroquinolone-associated disability (FQAD) and the lack of effective FQAD treatment, the drug should be used exclusively for serious infections [29]. The overwhelming potential side effects of antibiotics have triggered many scientific professionals and agencies to reassess the uses of antibiotics.

## 4. Livestock as a Major Contributor of Antibiotic Resistance

**Figure 3:**
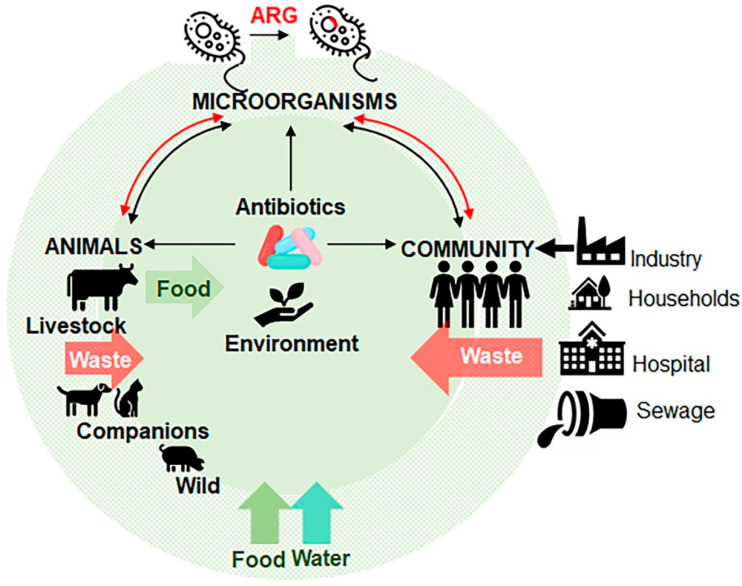
Schematics of the major route of antibiotic resistance genes (ARG, a red inserted line) dissemination in environment. The diagram indicates the contribution of human communities to the production of antibiotics and their uses in hospitals, farms, and households. Generated antibiotic waste is released onto sewage, hence contaminating water, soil, and environment. Bacteria develop ARG mutations as a result of such exposure to antibiotics in the environment, and in human and animal hosts. ARG-containing bacteria spread in humans and animals through direct infections, food, or environment. The arrows indicate the putative transmission paths of entry of antibiotics and ARG.

Animal livestock is an integral component of the global economy as a major contributor of food and materials, as well as draft power for transportation and agriculture operations in developing countries. To promote growth and weight gain, entire herds or flocks of farm animals are routinely fed with low dosages of antibiotics in their food or water. This practice is implemented to stave off disease in animals living in often crowded and unsanitary spaces. This activity leads to massive accumulation of antibiotics in the environment, and acquisition of antibiotic resistance in microorganisms coming in contact with an antibiotic [30] (Figure 3). Antibiotic consumption in the livestock sector is the highest in China (23%), the US (13%), Brazil (9%), and India (3%), accounting for the majority of worldwide sale of antibiotics [31,32,33]. The spread of antibiotic-resistant microorganisms to humans is carried through the consumption of contaminated food and drinks, direct contact with animals, or by environmental exposure, for example, through consumption of contaminated water (Figure 3). Both animal and human pathogens serve as donors of ARG to pathogens that infect humans. Table 3 summarizes major bacteria classes originating from animal species [34]. The use of fluoroquinolones (e.g., enrofloxacin) in food-producing animals has contributed to the spread of ciprofloxacin-resistant *Salmonella*, *Campylobacter* and *E. coli*, which are resistant to most therapies. A global trade with animal products contaminated with ARG affects the food supply in new regions. For instance, the use of a glycopeptide (avoparcin) as an antibiotic and a growth promoter in animals in Europe resulted in the expansion of vancomycin-resistant enterococci (VRE) in commensal microorganisms in livestock, on meat from these animals, and in the commensal flora of healthy humans worldwide [35].

To decrease global antibiotic-resistant bacterial infections, some measures have been implemented with respect to the use of antibiotics for non-therapeutic purposes, such as antibiotic use in animals intended for food production [36]. The imposed ban on the use of avoparcin in animal feed in the European Union has reduced the incidences of VRE in animals and its occurrences in the general population [37]. The efficacy of these measures suggests that animal-derived ARG could be one of the major sources for development of antibiotic resistance. Substantial attention is focused on the understanding of molecular mechanisms involved in the human acquisition of ARG from the animals. The transfer of antibiotic resistance determinants from animal to human through horizontal gene transfer is extremely difficult to detect and quantify. It is thought to drive the evolution of metallo-β-lactamase, e.g., NDM-1, and perhaps the use of antibiotics in agriculture accelerated this process [24,25,38]. The ARG phenomenon greatly hinders the progress of agriculture.

**Table 3:** ARG in animal production settings.

Sl. No.	Bacterial Species	Infection	Antibiotic Resistance Pattern	Sources of Human Infection	Genes
1	*Campylobacter* spp.	Gastrointestinal sequelae: Guillain-Barré syndrome	Fluoroquinolones, erythromycin	Food-producing animals (poultry)	*tetO, gyrA* [39,40]
2	*Enterococcus* spp.	Sepsis, urinary tract	Aminoglycosides ampicillin vancomycin	Food-producing animals (poultry); People exposed to hospital care, food animals	*Tuf, VanC-1, VanC-2-VanC-3, pbp5* [41,42,43,44,45]
3	*E. coli*	Gastrointestinal, urinary tract, diarrhoea	Quinolones sulphonamides trimethoprim	Childcare facilities	*Bla, qnrS, frdD* [46,47,48]
4	*Salmonella* spp. (non-typhoidal)	Gastrointestinal, diarrhoea	Cephalosporins quinolones tetracyclines	Food-producing animals (pigs, cows, poultry)	*IntI1, qnrA* [49,50,51,52]
5	*S. pneumoniae*	Otitis media, pneumonia, sinusitis, meningitis	Penicillin, macrolides, cephalosporins, tetracyclines	Childcare facilities, paediatric populations	*erm(B), mef* [53,54,55,56]
6	*S. pyogenes*	Pharyngitis, impetigo, cellulitis	Macrolides, tetracyclines	Childcare facilities, paediatric Populations, schools	*ermB, ermA and mefA* [57]
7	*S. aureus*				
	Community-associated	Skin, soft tissue, pneumonia, sepsis	Methicillin, cephalosporins, macrolides	Childcare facilities, injections, drug users	*erm(A), erm(C), tetK, tetM, aacA-aphD, vat(A), vat(B) and vat(C)* [58,59]
	Healthcare-associated	Endocarditis, pneumonia, sepsis	Methicillin, cephalosporins, quinolones, aminoglycosides, macrolides	People exposed to healthcare facilities such as nursing homes, dialysis, recent surgery or hospitalization	
8	*N. gonorrhoeae*	Urethritis, pelvic inflammatory disease	Penicillin, cephalosporins, quinolones	Commercial sex workers	*penA, penB, NorM* [60,61]

## 5. Scale of Antibiotic Use in Animals and Humans

Global use of antimicrobial substances in animal production for food (milk, eggs, and meat) was estimated at 63,151 ± 1560 tons (100%) in 2010, with a projected increase to 105,596 ± 3605 tons (167%) by 2030. The additional 34% rise will depend on the implementation of intensive farming systems by 2030. A recent study provides a projection of antibiotic use for livestock in India, where quinolones are expected to increase up to 243% by 2030, compared to their use in 2015, while the use of ampicillin and co-trimoxazole has declined [62]. It is envisaged that by 2030, the consumption of antimicrobials in Asia could reach roughly 51,851 tons; representing 182% of the current global consumption of antimicrobials in animal food in 2010. An overall 176% increase in antibiotic use was observed during the decade 2000–2010 in Brazil, Russia, India, China, and South Africa (BRICS) [63]. Animal consumption of antimicrobials in BRICS countries is expected to increase up to 199% by 2030 compared to current use. In human populations its expected growth will be around 113% during the same period [64]. India’s consumption of 12.9 × 10^9^ units of antibiotics (10.7 units/person) made it the largest consumer, followed by China, which used 10.0 × 10^9^ units (7.5 units/person) in 2010. The United States used 6.8 × 10^9^ units (22.0 units/person) of antibiotics during this time [32]. BRICS countries are five major rising national economies. From 2000 to 2010, antibiotic sales in the health care sector in India and China increased to 123% and 157% respectively [63]. This intensified antibiotics production significantly pollutes the environment.

## 6. Anthropogenic Contamination of Environment with Antibiotics and ARGs

Antibiotics can enter the environment through different routes (Figure 3). Antibiotics produced by industry as well as their metabolites are released from plants, hospitals, farms, and households with biological wastes (urine, faeces, sputum, placenta, tissues and organs) or by means of abandoned animals (e.g., cattle in India), stray animals (dogs, pigs, and birds) and open human defecation in slum areas. From the sewage, waste water treatment plants (WWTPs), and surface run off the antibiotics and/or ARG contaminate water and can be dispersed on fields that directly or indirectly enter humans’ and animals’ food chain systems [23,65,66]. The resistant bacteria follow similar routes to invade human systems [67]. These routes result in an environment where antibiotics, ARGs, antibiotic resistant bacteria, and the environmental bacterial flora can interact. These types of environments become likely a hotspots for the development of new ARGs by horizontal gene transfer that cross-contaminate different animal species. Humans come in contact with resistant microorganisms through numerous routes including consumption of contaminated foods, interactions with animals, and within contaminated environments. Infected human hosts spread ARGs to microflora inhabiting the hosts [23] and within communities (Figure 3). For instance, the β-lactamase cblA present in *Bacteroides* is one of the most abundant ARGs in the microbiota of both healthy persons and patients [68,69]. The progress and challenges in the understanding of ARG in the microbiota have been described in numerous excellent reviews [70,71,72,73,74].

Metagenomics is a diagnostic tool for detection of pathogens outbreaks in the faecal samples and tracking ARG in individual patients, which is known as resistome profiling [71,75,76]. Future advances in genome sequencing technologies are likely to facilitate high-throughput characterization of the resistome by metagenomic sequencing of microbiome in patients and assessing the possibility for horizontal gene transfer.

## 7. Alternative Strategies to Combat Antibiotic Resistance

Alternative strategies are imperative to combat infectious pathogens containing ARG [14,77]. The emerging therapies, including bacteriophage therapy [78], predatory bacteria, immunotherapeutics, haemofiltration devices, quorum sensing inhibitors, antimicrobial adjuvants, faecal microbiota transplantation (FMT), nano-antibiotics and nitric oxide (NO)-releasing nanoparticles, antimicrobial peptides (AMPs) or bacteriocins [15,79,80], essential oils, as well as competitive exclusion of pathogens through genetically modified probiotics and postbiotics, RNA therapy, and use of vaccines, are the prospective strategies discussed below.

### 7.1. Phage or Bacteriophage Therapies

Bacteriophages are viruses using bacteria as a host. They are extensively investigated as a replacement for antibiotics against drug-resistant pathogens despite numerous challenges [81,82,83]. Phage therapy was introduced in the early 1920s and in Georgia, Eastern Europe [84]. The technique is gaining popularity because phages are ubiquitous, harmless, and could be administered orally with food [84], topically on open wounds or surface infections [85], or intravenously during systemic infections (Table 4). The recent innovations in the gene therapies have created novel opportunities too for phage therapy to disrupt antibiotic resistance genes by Clustered Regularly Interspaced Short Palindromic Repeat (CRISPR) interaction with CRISPR-associated (Cas) (CRISPR/Cas) gene editing tool [86] or deliver antimicrobial proteins in recombinant phages [85]. However, the fine specificity of phage towards host bacterium species precludes their applications as an empiric therapy for acute infections. The phage libraries need a continuous update to ensure their efficacy against antibiotic resistant bacteria. Developing and establishing a complete library of phages for every plausible infectious bacterium is challenging [85].

### 7.2. Predatory Bacteria

Obligate predatory bacteria, including *Bdellovibrio bacteriovorus* and *Micavibrio aeruginosavorus*, populate soils and water globally [87]. Epibiotic or endobiotic predatory bacteria are attached to the membrane of the Gram-negative bacteria to consume prey contents and divide outside or inside their prey [88,89,90]. Several studies have demonstrated that *B. bacteriovorus* can kill more than 100 human bacterial pathogens acting as “living antibiotic” [91]. Predatory bacteria are free-living, are not pathogenic to humans, and have low immunogenicity. The use of *B. bacteriovorus* is being investigated as the novel therapeutic approach against antibiotic resistant and/or unidentified microbial infections. However, further research is required to understand the mechanism of predator–host interactions.

**Table 4:** Phage therapy in humans and in animal models.

Causative Agent	Model/Route	Condition	Type of Phage	Result
*Shigella dysenteriae*	Human/Oral	Dysentery	Cholera Bacteriophage	Recovered after 24 h [92]
*P. aeruginosa*	Murine/Oral	Sepsis	Phage strain KPP10	66.7% mortality reduction [93]
*Vancomycin-resistant E. faecium*	Murine/Intraperitoneal injection (i.p.)	Bacteremia	Phage strain C33 & ENB6	100% mortality reduction [94]
*C. difficile*	Hamster/Oral	Lleocecitis	Phage strain 135I	Prevented infection [95]
*Vibrio cholera*	Human/Oral	Cholera	Cholera Bacteriophage	93% survival in treated group vs. 37% in control group [92]
*Imipenem-resistant P. aeruginosa*	Murine/I. p. injection	Bacteremia	Phage strain Ø9882	100% mortality reduction [96]
*B-lactamase producing E. coli*	Murine/I. p. injection	Bacteremia	Phage strain Ø9882	100% mortality reduction [96]
*S. aureus*	Rabbit/Subcutaneous injection	Wound Infection	Phage LS2a	Prevented infection [97]
*Salmonella Typhi*	Human/Oral	Typhoid	Pyophage, Intestiphage, Staphylococcal bacteriophage, PhageBioDerm	5 fold decrease in typhoid incidence compared to placebo [98]
*MDR S. aureus*	Human/Tropical	Diabetic foot ulcer	Staphylococcal Phage Sb-1	100% recovery [99]
*Antibiotic-resistant P. aeruginosa*	Human/Oral	Chronic otitis	Biophage-PA	Improved symptoms in double-blind, placebo-controlled phase I/II trial [85]
*E. coli*	Murine/I. p. or subcutaneous injection	Meningitis and sepsis	Lytic Phage EC200PP	100% and 50% mortality reduction meningitis and sepsis, respectively [100]

### 7.3. Immunotherapeutics

Immunotherapeutics are biomolecules that improve immune responses in the host against infectious agents. A large number of immune adjuvants such as cytokines interleukin-2 (IL-2), IFN-gamma, IL-7, IL-12, as well as granulocyte macrophage colony stimulating factor (GM-CSF) and programmed cell death ligand-1 antibody are under clinical investigation to improve hosts’ immune defence in subjects with antibiotic resistance or immunocompromised patients [101,102]. For instance, G-CSF stimulates neutrophil production in patients with low neutrophil counts caused by chemotherapeutics. Pegfilgrastim is most widely used synthetic immunostimulant of G-CSF production [103]. In agriculture, a bovine G-CSF or its inducer pegbovigrastim are administered to cattle prior to parturition to boost the immune system and decrease the incidence of mastitis [104,105,106].

Monoclonal and polyclonal antibodies provide passive immunity against bacterial pathogens. The antibodies mAb F598, recognizing the major component of bacterial Gram-positive and Gram-negative cell wall poly N-acetylglucosamine (PNAG), are in a phase I clinical trial [107]. The two neutralizing, human monoclonal antibodies against *C. difficile* toxins A (CDA1) and B (CDB1) reduced the recurrence of *C. difficile* infection in double-blind placebo randomized controlled studies [91,108]. Recently, human monoclonal antibody bezlotoxumab was approved by US Food and Drug Administration (FDA) for prevention of recurrent *C. difficile* infection [109]. The advantageous immune response against infections requires precise timing for intervention with immunotherapeutics that could limit its applications.

### 7.4. Haemofiltration Devices

Extracorporeal pathogen removal filters such as mannose binding lectins [110] or bound heparin [111] are being studied. These therapies can bind and remove an array of blood stream pathogens. These haemofilters will cause reduction in the bacterial load which allows the host to develop innate and adaptive immune responses against residual antibiotic-resistant pathogens.

### 7.5. Quorum-Sensing Inhibitors

Bacteria behave as single organisms at low densities in a favourable environment. However, they acquire multicellular type of communication at high density or in adverse, antimicrobial environments by signalling termed quorum sensing (QS). Bacterial QS molecules include:

(1) Oligopeptides (5–10 amino acid cyclic thiolactone), such as N-acyl homoserine lactones used by Gram-negative bacteria [112,113],
(2) Furanosyl borate (Autoinducer-2, AI-2),
(3) N-acyl homoserine lactones (AHLs),
(4) Methyl-dodecanoic acid, and
(5) Hydroxyl-palmitic acid methylester [114,115].

In response to QS molecules, bacteria express numerous genes mediating bioluminescence, virulence, biofilm formation, sporulation, and other processes. Two widely studied QS molecules are AHL and peptides used by Gram-positive bacteria. The substances which inhibit the signal transduction and virulence activities of bacteria [116,117,118] are termed QS inhibitors, quorum quenchers, or antipathogenic signal interference. A recent study has identified 4-aminoquinolone as QS inhibitor in *S. marcescens and P. auroginosa* [119]. Recent studies have identified the range of new QS inhibitors derived from different sources, such as ajoene, iberin, sulforaphane, phenolics, O-glycosylated flavanones, polyphenols, urolithins, limonoids, caffeine, *Chamaemelum nobile* flower extract, leaves extract from Kalanchoe (*Bryophyllum pinnatum*), phytols, avellanin C, pigments (melanin, melanoid, pheomelanin), cyclic dipeptides, quercetin, engineered variant of hyper-thermostable lactonase SsoPox, thermostable lactonase, and colostrum hexasaccharide. These QS inhibitors have been used against human pathogens such as *P. aeruginosa*, *Yersinia enterocolitica*, *Aeromonas hydrophila*, *S. aureus*, *Chromobacterum violaceum*, *A. baumannii*, and *E. coli* [120]. QS inhibitor gallium effectively controls a biofilm formation via inhibition of iron metabolism [119,121,122,123,124]. The application of QS inhibitors for disruption of biofilms is being investigated for applications improving outcomes in systemic infections [125,126].

### 7.6. Antimicrobial Adjuvants (AA)

AA modify the efficacy of existing antibiotics without changing theirintrinsic antimicrobial activity. AA reverse the bacterial mechanisms of antimicrobial resistance [127,128,129]. The antibiotic efficacy is modified by any of the following mechanisms [130,131,132,133,134,135,136,137,138].

(a) Biofilm disruption.
(b) Augmenting the uptake of antimicrobial in the target cell.
(c) Enhancing the oxidative stress in bacteria.
(d) Supressing the ARG.
(e) Inhibition of bacterial efflux pumps.

Different antimicrobial adjuvants classes such as efflux pump inhibitors (e.g., quinolines,), β-lactamase inhibitors (e.g., clavulanic acid), membrane permeabilizers (e.g., aminoglycosides), antivirulence compounds (e.g., OASS-inhibitors, SAT-inhibitors, Cys-inhibitors) have been used against Gram-negative and Gram-positive bacteria. The oral pharmaceutical Augmentin contains β-lactamase inhibitors clavulanic acid and amoxicillin. It effectively treats a wide range of bacterial infections, including bronchitis and Lyme disease [139]. Recent study has demonstrated that efflux pump inhibitors, including N-acetylcysteine, Tris-EDTA, and disodium EDTA have intrinsic antimicrobial activity and overcome antibiotic resistance. These AA could be used to enhance the efficacy of existing antibiotics against Gram-negative and multidrug-resistant bacteria [140,141]. Thus, AA provide an economical alternative to time-consuming and costly development of new antibiotics targeting antibiotic resistance.

### 7.7. Faecal Microbiota Transplantation (FMT)

**Figure 4:**
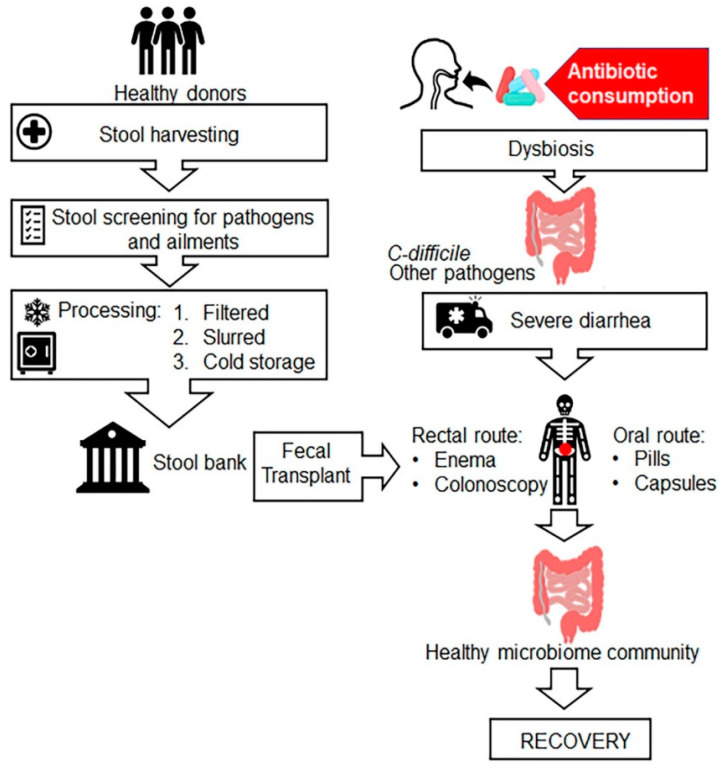
Schematic illustration of faecal microbiota transplantation (FMT) procedure. The left panel of the above figure depicts sample preparation where stool is harvested from healthy donors, processed via different stages such filtration, slurry preparation followed by cold storage in stool bank. The right panel illustrates the FMT procedure, where processed faecal microbiota of healthy donor stored in stool bank is either delivered via rectal route or oral route to the diseased patients (recipient) to provide a healthy microbiome community.

FMT is also known as faecal bacteriotherapy, faecal transfusion, faecal transplant, faecal enema, human probiotic infusion (HPI) and stool transplant. FMT is the process of transplantation of bacterial solution from faecal matter of a healthy individual donor into a recipient’s intestinal tract for total restoration of gut microbial flora using various methods including enema, nasogastric, nasoduodenal and colonoscopic routes (Figure 4) [142,143]. In veterinary medicine, it is known as “transfaunation” treatment for ruminate animals [144]. FMT was first introduced by Ben Eiseman and colleagues in 1958 for the treatment of four patients with pseudomembranous colitis [145], although the use of faecal enema therapy was described by Ge Hong in fourth-century China [146]. Few studies have shown that FMT is an effective treatment for people with *C. difficile* infection along with other gastrointestinal diseases, such as irritable bowel syndrome (IBS), colitis, constipation, diarrhoea, several neurological conditions such as Parkinson’s and multiple sclerosis [147]. FMT is successfully used in clinical practice for treatment of recurrent *C. difficile* infection that cannot be cured with antibiotics. Currently, different microbiota-based products for other diseases are under development and/or in clinical trials [148]. Ethical issues appear to be another hindrance despite FMT safety and efficacy. Further research is needed to advocate for efficacy of FMT therapy against global antibiotic resistance menace.

### 7.8. Nanoantibiotics

**Figure 5:**
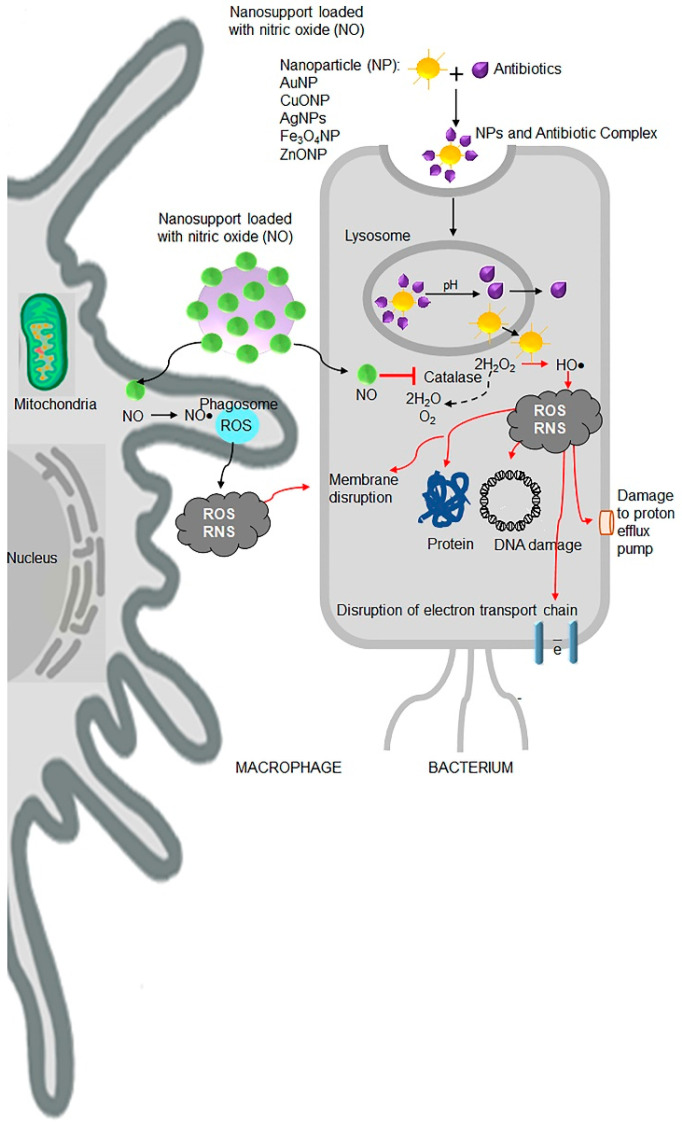
Schematic principle of action by nano-antibiotic therapies including nitric oxide (NO) releasing nanoparticles and nanoparticles in combination with antibiotics. The various nano-antibiotics (nAbts, purple shape) and NO-releasing nanoparticles (green circles) act via two components: metal ions (Ag^+^, Cu^2+^, Zn^2+^) (yellow particle in the centre) and a releasing component such as NO with or without antibiotics. Both components increase production of reactive oxygen species (ROS) in the bacterium as well as in the host immune cells (e.g., macrophages, neutrophils). Left panel, in the bacterium, NO inhibits catalase activity, which leads to rise in levels of hydrogen peroxide. In the presence of transition metals of nanoparticles catalyse conversions of hydrogen peroxide to a hydroxyl radical (HO•). Hydroxyl radical is one of many ROS and RNS responsible for oxidative and nitrosative stress and death of bacteria. ROS leads to disruption of cell membrane, interruption of transmembrane electron transport, oxidation of cellular components, protein and DNA damage. These actions disrupt structural and functional integrity of bacteria.

Although bacteria develop resistance to ‘free’ antibiotics, such as amphotericin B, oxacillin, cloxacillin, amoxicillin, cephalexin, cefotaxime, ceftazidime, vancomycin, streptomycin, and erythromycin, the coating of antibiotics on metal nanoparticles show enhanced antibacterial, antiviral, and anticancer efficacy (Table 5). Various research groups have demonstrated antimicrobial efficacy of silver (Ag) [149], copper (Cu) [150], gold (Au) [151], titanium (Ti) [152] and metal oxide-based nanoparticles such as titanium dioxide (TiO_2_) [153], copper oxide (CuO) [154], zinc oxide (ZnO) [155], manganese oxide (MnO_2_), aluminium oxide (Al_2_O_3_) [156] with and without antibiotics. Nanoantibiotics are regarded as promising therapeutic candidates for future applications to combat antibiotic resistance in biomedical sciences (Figure 5) [157,158].

Nitric oxide (NO) is a potent agent against a wide range of Gram-positive and Gram-negative bacteria. NO is endogenously produced by oxidation of L-arginine to L-citrulline by NO synthase enzymes in eukaryotic cells [159,160,161]. Administration of exogenous NO donors or NO-releasing nanomaterial releases high concentrations of small gaseous molecules that permeate membranes (Figure 5). In bacteria, NO leads to the production of harmful ROS and reactive nitrogen species (RNS), such as peroxynitrite, dinitrogen trioxide (N_2_O_3_), and nitrogen dioxide (NO_2_), by mechanisms involving inhibition of catalase activity [162]. Both ROS and RNS are also produced in host macrophages and other immune cells pathways to destroy the microorganisms [163,164]. A study had reported that NO-releasing nanomaterials decreased the biofilm-infected wounds that promoted wound closure [165]. Thus, NO donor nanomaterial represents a new promising strategy to combat antibiotic resistance in the future.

**Table 5:** Effect of metal/metal oxide nanoparticles with or without antibiotics against various bacteria.

**S.No.**	**Metal Nanoparticles Used**	**Action against Bacteria**
1	Silver	*E. coli, M. tuberculosis,* MRSA, *S. aureus, S. pyogens, K. pneumonia,* [166,167,168,169,170,171,172,173,174,175].
2	Titanium	*K. pneumonia, S. aureus, A. baumannii, E. coli, Morganella morganii* [152]
3	Gold	MRSA, *E. coli, P. aeruginosa, S. aureus, Enterococcus* spp., *B. subtilis* [151,176,177]
**S.No.**	**Metal Oxide Nanoparticles Used**	**Action against Bacteria**
1	Zinc oxide	MRSA, *Streptococcus agalactiae* [155]
2	Manganese oxide	MRSA [178]
3	Manganese oxide	*E. coli* [156]
**S.No.**	**Metal and Metal Oxide Nanoparticle Composite Used**	**Action against Bacteria**
1	Zinc doped copper oxide nanocomposite	MRSA, *E. coli* [154]
2	Copper doped zinc oxide nanocomposite	*E. coli, S. aureus* [179]
**S.No.**	**Metal Oxide Nanoparticles in Combination with Antibiotics Used**	**Action against Bacteria**
1	ZnO and Antibiotics (cefotaxime, ampicillin, ceftriaxone, and cefepime)	*E. coli, K. pneumoniae, Sphingomonas paucimobilis, and P. aeruginosa,* respectively [180]
2	TiO_2_ nanoparticles in combination with antibiotics (β-lactams, cephalosporin, glycopeptides, aminoglycosides, flouroquinolones, azlides, macrolides, lincosamides, and sulphonamides)	Showed improved antibacterial activity [181]
**S.No.**	**Metal Nanoparticles in Combination with Antibiotics**	**Action against Bacteria**
1	Gold nanoparticles and Ampicillin	MDR *P. aeruginosa, E. aerogenes*, and MRSA [182]
2	AgNPs with ciprofloxacin, imipenem, gentamycin, trimethoprim, and vancomycin	MDR *E. coli, P. aeruginosa, E. faecalis, S. aureus, Micrococcus luteus, A. baumannii, K. pneumoniae,* and *Bacillus* spp. [183]

### 7.9. Plant-Derived Antimicrobials and Essential Oils

Historically, plant extracts have been used as an antibiotic in food preservatives. Plant-derived antimicrobials such as nerolidol, apritone, and bisabolol exert antimicrobial action combating Gram-positive and Gram-negative bacteria [184,185,186]. Moreover, no side effects and antimicrobial resistance toward these plant-derived phytochemicals have been documented thus far, probably, due to their multiple mechanisms of action. Essential oils are another type of secondary metabolites of aromatic plants. Liquid and volatile essential oils have significant medicinal properties in infectious and non-infectious diseases (Table 6) and have a low risk of antibiotic resistance [187,188]. Multiple studies, reviewed in [189], have revealed potent activity of essential oils from *Eucalyptus camaldulensis* against Gram-positive and Gram-negative bacteria. The complex composition of different essential oils and their specificity against different types of bacteria are now subject of intense investigation [190]. Pharmaceutical development use antimicrobials produced in flora, fauna, and microorganisms living in various ecological niches, including deep oceans, rain forests, and soils [191,192,193,194]. Potential use of natural antimicrobial metabolites is a promising strategy for controlling antibiotic resistance development in microorganisms in the future.

### 7.10. Probiotics, Postbiotics and Synbiotics

Probiotics are the alive microorganisms or microbial feed supplements. They primarily comprise two classes of lactic acid-producing microorganisms: the bifidobacteria, and lactic acid bacteria (LAB). These microorganisms include species of *Enterococus*, *Lactobacillus*, *Lactococcus*, *Pediococcus*, *Vogococcus*, *Aerococcus*, *Carnobaterium*, *Streprotococcus* and *Weisella.* Most LAB, due to their safe (GRAS) status, and the abundance of some genera in the GI tract, mammary gland and female genitourinary tract, are regarded as alternative health-promoting treatments [195]. Postbiotics are functional bioactive compounds such as short-chain fatty acids, teichoic acid and other fermentation products. Identification of novel animal origin probiotics, postbiotics, and the non-viable microbial probiotics or probiotic metabolites that have biologic activities in host [196,197,198,199] may facilitate the development of alternative therapeutic combinations. These adjuvants can improve dosing regimens of traditional antibiotics and lessen the burden of enteric infections and side effects of antibiotic therapies.

**Table 6:** Effect of essential oils against bacteria.

S.No.	Essential Oils (Components)	Active against Bacteria
1	Mentha (menthol, isomenthone, limonene, iso-menthanol, menthol acetate, carvone, β-pinene, α-pinene, 1,8-cineole, α-terpineol, isopulegol, pulegone, piperiton, piperitone oxide, and β-phellandrene.)	*S. aureus, Staphylococcus epidermidis, B. cereus,* and *E. coli, S. pyogenes, P. aeruginosa, Pseudomonas fluorescens, C. albicans,* and *V. cholerae,* [200,201,202]
2	Basil (Linalool, epi-α-cadinol, α-bergamotene, γ-cadinene, germacrene D, camphor. methylchavikol, methylcinnamat, linolen, eugenol, cis-geraniol, 1,8-cineole, α-bergamotene, β-caryophyllene, viridiflorol.)	*S. aureus* and *B. subtilis, Staphylococcus, Pseudomonas,* and *Enterococcus genera, L. monocytogenes* and *B. cereus Vibrio* spp. and *Aerobacter hydrophila* [203,204,205]
3	Oregano (thymol, carvacrol, ρ-cymene, thymoquinone, and γ-terpinene.)	*Sarcina lutea, S. aureus, C. albicans, E. faecalis,* and *B. cereus* [206,207]
4	Rosemary (α-pinene, myrcene, 1,8-cineole, camphor, camphene, α-terpineol, and borneol.)	*S. epidermidis, S. aureus, B. subtilis, Proteus vulgaris, P. aeruginosa,* and *E. coli.* [208,209,210,211]

### 7.11. RNA Therapy

The bacterial small (50–500 nucleotides) regulatory RNAs (sRNAs) participate in many events such as growth, virulence onset, biofilm formation, stress response and antibiotic resistance. Modulation of bacterial sRNAs function by specific drugs could enhance the efficacy of antibiotics [91]. Acquired bacterial immunity based on CRISPR/Cas interaction has been used to target extended spectrum beta-lactams, carbapenems, or colistin resistance genes without changes in the host microbiota [212].

These RNA-based therapies, such as sRNAs and RNA-guided CRISPR/Cas technologies holds promise for successful delivery of highly effective RNA elements into the bacteria to fight against antibiotic resistance.

### 7.12. Development and Use of Vaccines

Vaccines provide possible solutions for the emerging antimicrobial resistance (AMR) crisis. Vaccines continue to be one of the most effective interventions against primary and secondary antibiotic resistant bacterial infections. Several candidate vaccines against the most common bacteria, e.g., *C. difficile* (Phase III)*, M. tuberculosis* (Phase II)*, Group B Streptococcus* (Phase II)*, S. aureus* (Phase II)*,* are in mid-stage clinical development by major pharma companies [213].

## 8. Mitigation Steps to Curb the Menace of Antibiotic Resistance

Tackling antibiotic resistance with preventive protective measures and policies in combination with effective medicines is a high priority to ensure prevention and treatment of infectious diseases [214,215]. The following measures have been proposed:

- Strengthening of surveillance data.
- Improving awareness of antibiotic resistance.
- Improving the practices of antibiotic prescription.
- Improvement of poor sanitation, malnutrition, and endemic infections.
- Optimizing the use of antimicrobial medicines and restricting over the counter sale of antibiotics.
- Improving the public awareness and government commitment.
- Reducing the incidence of infection by various means.
- Reducing clinical trial risk.
- Boosting market value for not feeding animals antibiotics.
- Strengthening the regulation of farm feeding of antibiotics.
- Ensuring the quality of generic antibiotics.
- Early sharing of data.
- Organizing world antibiotic awareness week.
- Implementation of the global antimicrobial resistance surveillance system (GLASS).
- Establishing the global antibiotic research and development partnership (GARDP).
- Establishing the interagency coordination group on antimicrobial resistance (IACG).

## 9. Future Strategies, Challenges, and Outlooks

The biggest imminent threat caused by the spreading of antibiotic resistance is the rise of multi-drug resistant bacteria such as MRSA, VRE, and ESBL. Bacteria develop resistance to drugs by various resistance development routes, including the major spreading routes among bacteria by ‘Jumping DNA’ termed transposons. Barabas’s group have proposed a therapeutic new target, a transposase protein that blocks the transposon insertion mechanism between the bacteria and interrupts transfer of ARG [216]. Another promising innovation is an early detection technique for antibiotic resistance using a CeO_2_ nanoparticle biosensor. The search for better treatment strategies for antibiotic resistance is continuing. Combined chemical and biological approaches would contribute to the development of a new potent remedies to mitigate this immense threat.

The microbial infections posing threats to human and animal health with major antibiotic resistant pathogens challenging agricultural food supply and the integrity of the environment. While some examples of ARG dissemination between environmental and pathogenic bacteria are evident, the intricate mechanisms were described in a scarce number of studies remain incomplete Antibiotics became a part of modern medicine around seven decades ago, and their efficacy and safety do not meet the demands of the intensifying animal production and growing population facing global treat of infectious diseases. Experts from diverse fields such as clinical research, microbiology, genetic and computational engineering, imaging and modelling should work jointly to evolve strategies and develop novel therapeutics to address this problem.

## Figures and Tables

**Table 1:** Classification of antibiotic resistance threats.

Urgent	Serious	Concerning
1. *A. baumannii, P. aeruginosa*, carbapenem-resistant 2. *Clostridium difficile* (CDIFF) 3. *N. gonorrhoeae*-3rd generation cephalosporin-resistant, fluoroquinolone-resistant 4. Carbapenem- and 3rd generation cephalosporin resistant *Enterobacteriaceae*: *K. pneumonia*, *E. coli*, *Enterobacter* spp., *Serratia* spp., *Proteus* spp., and *Providencia* spp., *Morganella* spp.	1. *Streptococcus pneumonia*, penicillin-non-susceptible 2. *Haemophilus influenzae,* ampicillin-resistant 3. *Shigella* spp., fluoroquinolone-resistant 4. *Enterococcus* spp., vancomycin resistant 5. Multidrug-resistant *Acinetobacter* 6. Drug resistant *Campylobacter* 7.Extended-spectrum β-lactamase producing *Enterobacteriae* (ESBLs) 8. Multidrug-resistant *P. aeruginosa* 9. Drug-resistant non-typhoidal *Salmonella* 10. Drug-resistant *Salmonella serotype Typhi* 11. Drug resistant *M. tuberculosis* 12. Methicillin-resistant *S. aureus* (MRSA)	1. Group B *Streptococcus* (GBS), clindamycin resistant 2. Group A *Streptococcus* (GAS), erythromycin resistant 3. *S. aureus*, vancomycin resistant

**Table 2:** Mechanism of action of antibiotics.

Mechanism of Action	Name of Antibiotic Families
Inhibition of protein synthesis	Tetracyclines, aminoglycosides, streptogramins, ketolides, macrolides, lincosamides, daptomycin
Inhibition of DNA synthesis	Fluoroquinolones, daptomycin
Inhibition of RNA synthesis	Rifampin and other metronidazoles, daptomycin
Inhibition of cell wall synthesis	Penicillins, cephalosporins, carbapenems, monobactams, glycopeptides
Disrupt functions of bacterial outer membrane	Daptomycin, polymyxin B, colistin, and lipopetides
Competitive inhibition of folic acid synthesis	Sulfonamides, trimethoprim
